# Sentinel Lymph Node Biopsy Is Feasible in Dogs with Scars from Prior Local Excision of Solid Malignancies

**DOI:** 10.3390/ani12172195

**Published:** 2022-08-26

**Authors:** Elisa Maria Gariboldi, Damiano Stefanello, Mirja Christine Nolff, Donatella De Zani, Davide Zani, Valeria Grieco, Chiara Giudice, Camilla Recordati, Francesco Ferrari, Roberta Ferrari, Lavinia Elena Chiti

**Affiliations:** 1 Dipartimento di Medicina Veterinaria e Scienze Animali, Università degli Studi di Milano, 26900 Lodi, Italy; 2 Klinik für Kleintierchirurgie, Vetsuisse-Fakultät, Universität Zürich, CH-8057 Zurich, Switzerland

**Keywords:** scar, canine, cancer, sentinel lymph node

## Abstract

**Simple Summary:**

Sentinel lymph node (SLN) excision is gaining relevance in the management of various canine malignancies due to its recognized impact on staging and treatment choices. However, the technologies to perform SLN mapping are only available to a few referral centers, and there is increasing demand for secondary nodal staging after prior tumor excision at the primary care institution. This retrospective study investigated the feasibility and usefulness of SLN biopsy in dogs with surgical scars resulting from the removal of various solid tumors referred for further staging and/or adjuvant treatment options. Thirty-three dogs with 34 scars underwent SLN biopsy at a median of 50 days after primary tumor excision. An SLN was identified for 31/34 scars, translating to a detection rate of 91.2%. Metastases were identified with histopathology in 13/31 dogs (41.9%) and they all had an excision of a mast cell tumor. SLN biopsy should be suggested in dogs presenting with scars from prior solid tumor excision, considering the observed detection rate and the importance of knowing the metastatic status of the SLN in oncological diseases.

**Abstract:**

Sentinel lymph node (SLN) biopsy is a well-established staging tool in canine oncology. This study aims to explore the feasibility of SLN biopsy in dogs with scars from prior excised solid malignancies that were referred for further tumor staging and/or adjuvant treatment options. Mapping was either performed using radiopharmaceutical, methylene blue, and/or near-infrared fluorescent (NIRF) imaging. Thirty-three dogs with 34 scars from prior excision of the mast cell tumor (MCT) (*n* = 29), soft tissue sarcoma (*n* = 2), oral melanoma (*n* = 1), subungual melanoma (*n* = 1), and mammary adenocarcinoma (*n* = 1) were retrospectively enrolled. Primary treatment consisted of curative intent/wide tumor excisions in 50.0% of dogs and marginal excision in the remaining 50.0%. The median time between tumor excision and SLN biopsy was 50 days (range 17–110 days). The procedure was successful in 31/34 scars, translating to a detection rate of 91.2%. The SLN did not correspond to the regional lymph node in 19/31 scars (61.3%). SLN metastases were histologically identified in 13/31 (41.9%) dogs, all of them affected by MCT. Based on our results, SLN biopsy using lymphoscintigraphy/methylene blue and/or NIRF is feasible in dogs presenting with scars from the prior surgical excision of solid tumors, and should be suggested for accurate nodal staging.

## 1. Introduction

The sentinel lymph node (SLN), defined as the first lymph node on the drainage pathway of a tumor, may differ from the anatomically closest regional lymph node (RLN) in 22–63% of tumor-bearing dogs [1,2,3,4,5,6,7,8,9], and metastases to the SLN have been reported in up to 50–70% of dogs, depending on the tumor type [2,4,5,6,9,10,11,12]. Given the well-accepted impact of the accurate detection of nodal metastases on staging and treatment recommendations, the implementation of sentinel lymph node biopsy (SLNB) in the surgical management of canine malignancies has gained increased interest in recent years [8,13,14]. Various mapping techniques have been described to detect SLN in dogs, including lymphoscintigraphy and methylene blue, near-infrared fluorescence (NIRF) imaging, indirect lymphography with lipidic or aqueous contrast mediums, and contrast-enhanced ultrasonography (CEUS) [2,4,5,7,8,9,15,16,17,18]. Regardless of the technique used, the combination of a preoperative and intraoperative mapping method is recommended to increase the ability to correctly detect and excise the SLN [6,16]. However, the required technologies and human resources are currently not widely available in veterinary practices and only a few referral centers have access to SLN mapping techniques [6,7,16]. Consequently, dogs are increasingly presented to specialized centers for secondary lymph node staging after the primary tumor has been excised at the first-line care institution. However, SLN mapping in a scar comes with unique challenges. Independently of the mapping procedures, a peritumorally injected tracer (e.g., 99-m Technetium for scintigraphy, Indocyanine Green (ICG) for NIRF, and microbubbles for contrast-enhanced ultrasound (CEUS)) must be drained by the lymphatic network in order to reach the SLN. Previous surgical excision and postoperative inflammation can damage, disrupt, or increase the lymphatic ducts and drainage patterns, potentially leading to a failure in the mapping procedures [5,19,20]. The current body of literature is sparse and provides only contradictory information on the feasibility of SLNB in dogs with scar tissue from previous MCT excisions. Recently, a paper showed that SLNB failed in two dogs with a scar from a previous surgery, leading to questioning the feasibility of SLN mapping in the presence of scar tissue [5]. Conversely, SLN was successfully identified using radiopharmaceutical and CEUS in four and 24 cases, respectively [2,4]. 

To date, however, clinical studies specifically assessing the feasibility and success rate of secondary SLNB in dogs with scars from prior surgical excision of the primary tumor that have been referred for further staging and/or adjuvant treatment options are lacking. This study therefore aimed to investigate the feasibility and utility of SLNB in a cohort of dogs presenting with scars from previously excised solid malignancies. 

## 2. Materials and Methods

Clinical records of two teaching hospitals (University of Milan, Milan, Italy, and University of Zurich, Zurich, Switzerland) were reviewed for dogs with scars from previous solid tumors that were referred for further staging and/or adjuvant treatment options, and underwent SLNB between May 2017 and July 2022.

Dogs were excluded for the following reasons:

- Received other local treatment then surgical excision of the primary tumor, including radiotherapy or tigilanol tiglate injection
- Underwent prior excision of the RLN or had cytological evidence of metastases to the RLN
- Had cytologically or histologically confirmed local tumor recurrence at the time of SLNB.

At the time of SLNB, all owners had to sign written consent for the procedures and to data collection. All surgeries were performed for diagnostic (nodal staging) or therapeutic reasons (infiltrated margins) and in concordance with the national legislation for animal welfare.

Retrieved data included patient signalment (breed, sex, age, and bodyweight), the type and results of preoperative oncological staging, and the location and size of the surgical scar. The tumor type, grade (if relevant), and status of the excisional margins were also retrieved from the histopathological reports [21,22,23,24]. When available, the following variables were also retrieved: number and size of the primary tumor at the time of excision, anatomical location of the primary tumor, first presentation vs. recurrence, ulceration, type of surgical treatment performed at the primary care institution (curative intent/wide excision vs. marginal excision), the pattern of reconstruction after tumor excision (linear vs. non-linear) [25], and any healing complications that occurred at the site of the primary tumor removal. Excision of the primary tumor was considered with curative intent when the initial surgical report described the removal of 2–3 cm of lateral margins (or proportional margins in case of MCT < 3 cm of diameter [26,27]) and 1–2 deep fascial planes, or when the resulting surgical scar was at least three times larger than the longest diameter of the primary tumor. The remaining cases were considered marginal excision.

The time in days between the surgical excision of the primary tumor and SLNB was calculated. Sentinel lymph node mapping and extirpation were guided by preoperative lymphoscintigraphy and intraoperative gamma-probing and methylene blue at one institution (University of Milan), or either by intraoperative NIRF imaging only or in combination with lymphoscintigraphy at the other institution (University of Zurich).

Both mapping techniques were performed as previously described [9,11]. Briefly, either technetium-99 metastable labeled nano-sized human albumin or ICG (or both) were injected in two to four quadrants around the scar preoperatively. When radiopharmaceutical was used, planar regional static images were acquired with a gamma camera until the first draining lymph node was visualized, and a hand-held gamma probe was used intraoperatively to guide the surgical dissection towards the “hot lymph nodes”. Methylene blue was combined with a radiopharmaceutical to aid in the intraoperative identification of the SLN. For NIRF lymphography, a dedicated camera system (IC-Flow^TM^ or Visionsense VS3 Iridium^TM^) was used intraoperatively to identify the draining lymphatic ducts and lymph node, which was then dissected and excised under fluorescent guidance. Surgical resection of the scar was performed thereafter in case of previous infiltration or narrow excision of the primary tumor. The excised SLN(s) and surgical scar—in the case of revision—were submitted for histopathology. Lymph node status was reported as metastatic or non-metastatic; in the case of MCT, the histological classification by Weishaar and colleagues was used and SLN were categorized as HN0, non-metastatic; HN1, pre-metastatic; HN2, early metastatic; and HN3, overt metastatic [24]. SLN diagnosed as HN2 and HN3 were considered metastatic [24].

The mapping procedure was considered successful when surgeons were able to identify and remove at least one SLN. The information collected on SLN mapping and extirpation included the SLN mapping technique used; the number, size, and location of the SLN(s); the time for SLN extirpation (if available); correspondence between SLN and clinically expected RLN (identified based on the lymphosomes’ concept published by Suami et al., 2013) [28], and the histopathological status of the SLN. Postoperative complications at the lymphadenectomy site were also recorded.

The distribution of continuous variables (age, bodyweight, tumor size, scar length, time between tumor excision and SLN mapping, SLN size, and SLNB surgical time) was tested for normality using the Shapiro–Wilk test. The median, range, first quartile (Q1), and third quartile (Q3) were reported for non-normally distributed variables, and the mean and standard deviation were reported for normally distributed variables. Distribution of the categorical variables (breed, sex, primary tumor ulceration, tumor histopathological data, anatomical location of scars, type of excision and reconstruction, and histological status of SLN and of scars) was reported as the percentage of each modality on the total cases. Statistical analysis was performed with the software (R-Software vers R 4.2.1, packages rms and PASWR; www.R-project.org, accessed on 29 July 2022). The significance level was set at *p* ≤ 0.05.

## 3. Results

### 3.1. Sample Population

Thirty-three dogs with 34 scars from prior excisions of solid malignancies were included (Table 1). The breeds were distributed as follows: 12/33 (36.4%) mixed breeds; 4/33 (12.2%) Retrievers; 4/33 (12.2%) Jack Russell Terrier; 2/33 (6.1%) Boxers; 2 (6.1%) French Bulldogs; and 1/33 (3.0%) each of Epagneul Breton, Pug, Tibetan Terrier, Dachshund, Deutscher Pinscher, Maltese, Chihuahua, Australian Shepherd, and Cane Corso.

There were 13/33 (39.4%) intact males, 7/33 (21.2%) neutered males, 2 (6.1%) intact females, and 11/33 (33.3%) spayed females. The distribution of age (W = 0.891; *p* = 0.002) and bodyweight (W = 0.936; *p* = 0.046) were non-normal. The median age was 8 years (range 3–17 years; Q1: 7 years–Q3: 9 years) and the median bodyweight was 18.5 kg (range 2.4–55.0 kg; Q1: 10.6 kg–Q3: 29.9 kg).

The dimension of the primary tumor was available in all cases and was non-normally distributed (W = 0.696; *p* < 0.001); the median tumor’s longest diameter was 15 mm (range 4–100 mm; Q1: 10.0 mm–Q3: 24.5 mm). In 17/34 cases (50.0%), a curative-intent surgical excision was performed before referral, whereas in the other 17/34 cases (50.0%), primary local treatment consisted of marginal resection. One scar resulted from the excision of a local recurrent MCT, whereas all of the other scars were from the excision of tumors at first presentation. Ulceration was originally reported in 2/34 tumors (5.9%). In all cases, a linear reconstruction was performed after tumor excision, and no wound healing complications were reported. For the histopathological reports after primary treatment, the excised tumors were as follows 29/34 (85.4%) MCT (*n* = 19 cutaneous, *n* = 9 subcutaneous, and *n* = 1 mucosal/*n* = 17 Kiupel low grade, *n* = 2 Kiupel high grade); 2/34 (5.9%) soft tissue sarcoma (grade I and II); and 1/34 (2.9%) each of oral melanoma, subungual melanoma, and mammary adenocarcinoma (grade II). In 10/34 (29.4%) cases, surgical margins after primary treatment were tumor-free, in 21/34 (61.7%) they were infiltrated, and in 3/34 (8.8%) they were narrow. 

Distant metastases were excluded in 29/33 (87.9%) dogs. A complete preoperative staging consisting of abdominal ultrasound with fine-needle aspirates of the spleen and liver was performed in 24/33 dogs (72.8%) with MCT; of whole-body contrast-enhanced CT in 4/33 dogs (12.1%) with soft-tissue sarcoma (grade I), oral melanoma, subungual melanoma, and mammary adenocarcinoma; and of three-views thoracic radiographs and abdominal ultrasound in a dog (1/33–3.0%) with soft-tissue sarcoma grade I. In 4/33 (12.1%) cases with MCT (all cases were low grade), preoperative oncological staging was discussed with the owners, but was declined. 

The scar length reported in 33/34 cases (97.1%) was non-normally distributed (W = 0.813; *p* < 0.001) and the median value was 40 mm (range 10–200 mm; Q1: 18 mm–Q3: 65 mm). The anatomical location of the scars was 14/34 (41.3%) trunk, 6/34 (17.6%) proximal limb (above stifle or elbow), 6/34 (17.6%) genital or inguinal, 5/34 (14.7%) head and neck, and 3/34 (8.8%) distal limbs. The median time between tumor excision and SLN mapping was 50 days (range 17–110 days; Q1: 38.0 days–Q3: 66.5 days), with a non-normal distribution (W = 0.931; *p* = 0.032). Scar re-excision was concurrently performed in 24/34 cases (70.6%).

### 3.2. SLN Mapping and Biopsy

Sentinel lymph node mapping and extirpation were guided by radiopharmaceutical and methylene blue in 22/34 cases (64.7%), by NIRF imaging in 10/34 cases (29.4%), and by a combination of the two techniques in two cases (5.9%). A lymphatic drainage pathway was identified in 31/34 scars, leading to an SLN detection rate of 91.2%. In 3/34 scars (8.8%), the mapping procedure failed to identify lymphatic drainage and SLNs. In all three cases, SLN mapping was guided by a radiopharmaceutical and methylene blue at 65, 67, and 110 days after the excision of a cutaneous MCT (Table 1).

A total of 52 SLNs were removed, with a single SLN being excised in 14/31 cases (45.2%) and multiple (range 2–4) SLNs being excised from 17/31 scars (54.8%). All of the excised SLNs were either hot, blue, or fluorescent, or a combination of these. More specifically, a radiopharmaceutical and methylene blue guided the extirpation of 30 SLNs, of which 28 were hot and blue and two were hot, but failed to show blue coloration; all 17 SLNs removed under the guidance of NIRF imaging only were fluorescent; the five SLN removed with the guidance of a combination of a radiopharmaceutical, methylene blue, and ICG were positive to all tracers. The excised SLNs belonged to 40 sentinel lymphocenters, with 22/31 (71.0%) scars draining to a single lymphocenter and nine (29.0%) draining to two lymphocenters. The anatomical distribution of the SLN and lymphocenters are detailed in Table 1. The location of the SLN did not correspond to the RLN in 19/31 scars (61.3%). The size of the excised SLN was available in 29/54 SLNs (53.7%), with non-normal distribution (W = 0.903; *p* = 0.011) and a median longest diameter of 15 mm (range 5–40 mm; Q1: 11 mm–Q3: 19 mm). At the time of SLNB, 31 dogs had clinically normal RLNs, while two—one with an oral melanoma and one with a subcutaneous MCT—had an enlarged RLN. All enlarged RLNs (cytologically negative for metastases) were excised, and they all corresponded to the SLN. At histopathology, the SLN from the dog with oral melanoma was negative for metastases, whereas the SLN removed from the dog with MCT had early metastases (HN2 based on Weishaar et al., 2014).

Surgical time for SLN excision was available in 24 cases, and the distribution was normal (W = 0.970; *p* = 0.723) with a mean time of 36.5 ± 19.9 min. No complications related to the mapping procedure and no intraoperative complications of lymphadenectomy were recorded. Self-limiting postoperative complications at the lymphadenectomy site occurred in two cases, and consisted of seroma (*n* = 1) and hematoma (*n* = 1). 

Thirteen of 31 dogs (41.9%) in which the SLNs were identified had metastatic lymph nodes at the histopathological examination, and they were all affected by MCT (Table 1). If considering only the cases of MCT, the rate of SLN metastasis (HN2, early metastasis, plus HN3, overt metastasis, based on Weishaar et al., 2014) was 50.0%. Scar re-excision resulted in tumor-free margins in 22/24 cases and in infiltrated margins in 2/24 cases.

## 4. Discussion

This retrospective study investigated the feasibility and utility of SLNB in dogs referred with scars from prior excision of solid malignancies using lymphoscintigraphy/methylene blue and/or NIRF. In the study population at least one SLN was identified and excised in 31 out of 34 cases, leading to a detection rate of 91.2%. This detection rate is comparable to those reported in dogs undergoing SLNB and simultaneous first tumor excision [5,6,9,11,16]. Our findings therefore suggest that the procedure can also be successfully implemented in dogs that already had curative-intent or marginal excision of their primary tumor before.

In recent years, implementation of SLNB in the surgical management of various solid tumors in dogs has been advocated due to the recognized impact of accurate detection of nodal metastases on tumor staging [2,3,5,6,8,11,16]. The therapeutic benefits both of prophylactic lymphadenectomy (HN1) and excision of early and overt metastatic lymph nodes (HN2—HN3) [24] in dogs with integumentary MCT have further corroborated the importance of a guided surgical approach to the lymphatic basin [29,30,31,32]. However, specific investigations of the feasibility of SLN mapping and extirpation in dogs presenting with scars after primary tumor excision have not been previously performed. The available evidence is limited to a few studies including lymphatic mapping procedures of both primary tumors and surgical scars, with inconsistent results. Worley firstly described the use of radiopharmaceutical and methylene blue to guide SLNB in dogs with MCT and was able to successfully identify the lymphatic drainage of 4 scars from prior MCT excision measuring 10 to 35 mm [2]. Likewise, SLN mapping and excision guided by NIRF imaging were successful in seven dogs with scars tissue resulting from surgical excision of MCT in a recent study [9]. Finally, Fournier and colleagues reported that CEUS allowed for the identification of at least one SLN in 24 dogs presenting with scars from previously treated MCT, although the number of SLN identified in these animals was significantly lower than in those that did not receive previous surgery [4]. Conversely, in a recent investigation on a larger cohort of dogs, SLNB guided by radiopharmaceutical and methylene blue failed in two dogs presenting with local recurrence of MCT and in one dog that had a previous surgical approach to the lymphocenter identified as sentinel during preoperative lymphoscintigraphy [5]. This is consistent with the results of the present study, in which failure to identify a lymphatic drainage pathway was recorded in three dogs that underwent SLN mapping with radiopharmaceutical after that curative-intent excision of their MCT had been performed at the primary care institution. In two out of three patients, preoperative lymphoscintigraphy was unsuccessful, and owners denied any further intraoperative mapping as well as any surgical approach to the lymph nodes, while in the other case identification of preoperative drainage was uncertain, and intraoperative gamma-probing did not identify any SLN. In human medicine, in 58–78% of women presenting for SLN biopsy after previous breast cancer surgery, an SLN can be detected with intraoperative gamma-probing despite negative pre-operative lymphoscintigraphy, suggesting a lack of accuracy of the preoperative technique in these cases [33,34]. The combination of a preoperative and intraoperative mapping technique can improve the accuracy of SLNB in dogs undergoing surgical excision of the primary tumor and SLN simultaneously [6,16]. Of note, in the ten dogs where ICG was utilized as a sole method for SLNB, at least one SLN could be detected in all cases, and only in three dogs, this lymph node was categorized as HN0. Although large studies that evaluate accuracy of ICG mapping only are currently not available for dogs, this might indicate that the technique is by itself reliably to identify the SLN, as has been demonstrated in various human studies before [35,36]. However, future investigations are warranted in dogs to establish the accuracy and utility of preoperative SLN mapping techniques and clarify the impact of the combination of preoperative and intraoperative phases in dogs presenting with scars from prior surgery.

The feasibility and accuracy of delayed SLNB after previous surgical treatment has been widely investigated in humans with melanoma, genital tumors, and breast cancer [33,37,38,39,40,41]. Detection rates ranging from 85.5% to 100% are similar to those reported when concurrent tumor excision is performed and are comparable to the detection rate of 91.2% recorded in our sample population [33,37,38,39]. 

Several factors have been investigated that can potentially affect the accuracy of SLNB in humans that received prior tumor excision, including the extent of primary surgery, the time between primary surgery and SLNB and the use of complex skin reconstructive techniques [40,41,42]. In two recent studies on women with breast cancer, the size of initial lumpectomy had no effect on SLN identification rate [33,43]. The small sample size precluded the use of a statistical model for similar purposes in the present study. However, it should be emphasized that SLNB was successful in 16 out of 17 dogs that received marginal excision of the primary tumor, as well as in 15 out of 17 dogs that underwent previous curative-intent surgery. A failure occurred in two dogs with scars measuring 6.5 and 12 cm respectively and resulting from curative-intent excision, but also in one case after marginal excision of the primary tumor resulting in a 1.4 cm scar. Based on these observations, it seems reasonable to assume that SLN detection rate is not influenced by the extent of the primary surgery in these dogs in which a linear pattern of reconstruction has been applied, as previously recognized in humans. Further studies with a larger sample population are needed to statistically compare the SLN detection rate between widely and marginally excised tumors and to determine whether more extensive surgeries resulting in non-linear reconstruction can alter the lymphatic network.

Another variable that can potentially affect the detection rate of SLN in patients with surgical scars is the time elapsed between primary tumor treatment and lymphadenectomy. In women with breast cancers, available data are conflicting, with one study suggesting no impact of timing between lumpectomy and SLNB [43] and another investigation demonstrating a four times higher risk of failure when SLNB is attempted before 36 days after lumpectomy [33]. In the present study, the time between primary tumor excision and SLNB was extremely variable, ranging between 17 and 110 days. Again, the influence of timing on the detection rate was not statistically evaluated in this study due to the low number of unsuccessful procedures and consequent low power of statistical tests. In the present study, all procedures performed before 50 days after primary tumor excision were successful, and it seemed that a shorter timelapse does not negatively impact the accuracy of the technique. This observation is supported by a recent experimental study on healthy beagles, in which SLN mapping was successful in all included cases at 18 days after excision of a small skin area [19]. Further studies should elucidate the impact of timing between primary tumor excision and SLNB on the SLN detection rate in dogs 

Metastases in the SLN were diagnosed in 13 dogs, representing 41.9% of cases in which an SLN was successfully excised. All these dogs had a histopathological diagnosis of MCT, leading to an incidence of nodal metastases of 50.0% for dogs with MCT included in this study. The rate of nodal metastases that we report is comparable with what was previously reported after SLNB in dogs with MCT [2,4,5,8,11]. The fact that a metastatic lymph node was excised in nearly half of the dogs included in this study also underlines the importance of performing a surgical approach to the SLN in dogs that already had surgical excision of the primary tumor and that are referred for further staging and/or adjuvant treatment recommendations, especially in the case of MCT. This consideration holds particularly true when considering the therapeutic effect of the excision of early and overtly metastatic lymph node in cutaneous canine MCT [29,30,31,32].

One of the main limitations of the study presented here is the lack of a long-term follow-up that precluded the assessment of the false negative rate. A false negative occurs when nodal metastases are identified in a second or third echelon lymph node during follow-up, without previous evidence of metastases to the SLN. The false negative rate is one of the main factors affecting the accuracy of SLNB, and it has thus been widely explored in humans. It has been reported that complex skin reconstructive surgery, especially rotational flaps, can lead to severe alterations of the lymphatic drainage, hence resulting in a higher rate of false negative results when SLNB is performed in patients that had previously received this type of surgery as the first-line treatment [40,41,44]. Comparable studies are lacking in the veterinary literature, although a mismatch between preoperative and immediate postoperative lymphatic drainage has been reported in four out of eight experimental healthy beagles after surgical removal of a small skin area on the antebrachium [19]. Such an observation suggests that alteration of the original lymphatic drainage can indeed occur even after the removal of a small amount of tissue, and underscores the need for further investigation on the impact of previous surgery on the false negative rate of SLNB. Other limitations of the study are mainly related to its retrospective nature and to the relatively low size of the sample population and the paucity of failure procedures, which precluded the possibility to perform statistical analysis and to evaluate the impact of tumor variables (tumor type, size, and location), surgical variables (extent of surgery, time between primary excision, and SLN biopsy), and patients’ variables (age, sex, bodyweight, and body condition score) on the accuracy of SLNB in dogs with scars from prior tumor excisions.

## 5. Conclusions

In conclusion, this retrospective study shows initial results regarding SLNB guided by a radiopharmaceutical and methylene blue or NIRF imaging in scar tissue. Both mapping procedures are feasible in dogs with prior surgical excision of solid tumors. The detection rate and SLN metastases rate were comparable to those reported for simultaneous SLNB and tumor excision, especially in the case of MCT. Considering the recognized importance of SLN staging for optimal oncological management, dogs presenting with scars from previous surgery with linear reconstruction should not be excluded from SLNB. Further studies should collect data on the oncologic outcome and on the detection rate in scars from complex reconstructive skin surgery.

## Figures and Tables

**Table 1:** Signalment, histopathological data of excised tumors and SLN, and clinical characteristics of the scars, as well as of SLN mapping and extirpation in the study population.

Signalment	Tumor Type	Time to SLNB (days)	Scar Length (cm)	Prior Tumor Excision	Scar Location	Mapping Technique	RLN (Suami et al., 2013. [28])	SLN	SLNs Histopathology
Labrador R, SF, 8 years, 30.8 kg	MCT, Kiupel high grade	65	6.5	CI	L thoracic mammary	Radio + MB	Axillary L	No drainage identified at preoperative mapping	-
JRT, IF, 9.5 years, 8 kg	MCT, Kiupel low grade	110	12	CI	R thoracic mammary	Radio + MB	Axillary R	Drainage to accessory axillary at preoperative mapping; no SLN identified intraoperatively	-
Boxer, IM, 7 years, 36 kg	MCT, Kiupel low grade	67	1.4	Marginal	Inguinal fold R	Radio + MB	Inguinal R	No drainage identified at preoperative mapping	-
Golden R, IM, 3 years, 37 kg	MCT, Kiupel low grade	59	1	Marginal	L thoracic lateral	Radio + MB	Axillary L	Accessory axillary L	HN0
Corso Dog, IM, 5 years, 55 kg	MCT, Kiupel low grade	37	6	Marginal	II hand digit L	Radio + MB	Superficial cervical L	Superficial cervical L	HN1
Epagneul Breton, SF, 5 years, 13.9 kg	MCT, Kiupel low grade	58	2	Marginal	R inferior lip	Radio + MB	Mandibular R	Mandibular R	HN1
Mixed breed, IM, 7.6 years, 13.3 kg	MCT, Kiupel low grade	36	2.1	CI	II toe digit L	Radio + MB	Popliteal L	Popliteal L	HN2
Mixed breed, IM, 7 years, 10 kg	MCT, Kiupel low grade	17	4	CI	Median sternal	Radio + MB	Axillary R vs L	Accessory axillary L	HN2
Boxer, IF, 8 years, 25.3 kg	Subcutaneous MCT	37	1.5	Marginal	thoracic L	Radio + MB	Superficial cervical vs. axillary L	Axillary L	HN1
Mixed breed, NM, 8 years, 24.5 kg	MCT, Kiupel low grade	48	20	CI	L medial tight	Radio + MB	Popliteal L	Medial iliac L	HN0
Mixed breed, NM, 7 years, 49 kg	Subcutaneous MCT	99	8	Marginal	R lateral thigh	Radio + MB	Popliteal R	Inguinal R	HN2
Mixed breed, SF, 7 years, 18.5 kg	Subcutaneous MCT	78	1.5	CI	L inguinal mammary	Radio + MB	Inguinal R	Inguinal R	HN2
Labrador R, IM, 9 years, 42.5 kg	MCT, Kiupel low grade	34	1.8	Marginal	R preputial	Radio + MB	Inguinal R	Inguinal R and L	HN2
Mixed breed, IM, 12 years, 22.2 kg	Perivascular Wall Tumor	84	15	Marginal	L thoracic	Radio + MB	Axillary L vs. Accessory axillary L	Axillary L and Accessory axillary L	Negative
Mixed breed, IM, 17 years, 6.6 kg	Oral Melanoma	38	2	Marginal	L inferior lip	Radio + MB	Mandibular L*	Mandibular L	Negative
Tibetan Terrier, IM, 5 years, 11.3 kg	Conjunctival MCT	34	1	Marginal	L conjunctival fornix	Radio + MB	Mandibular L	Parotid L	HN0
Dachshund, IM, 10 years, 8.4 kg	Subungual Melanoma	45	1	Marginal	V hand digit R	Radio + MB	Superficial cervical R	Superficial cervical R	Negative
Mixed Breed, SF, 10 years, 29 kg	Soft Tissue Sarcoma	70	15	Marginal	R thigh	Radio + MB	Inguinal R	Popliteal R	Negative
French Bulldog, IM, 5.8 years, 13.1 kg	MCT, Kiupel high grade	40	6.5	CI	R sternal	Radio + MB	Axillary R	Axillary R and Accessory axillary	HN3
Labrador R, IM, 8.3 years, 39.8 kg	Subcutaneous MCT	60	9.5	CI	R cervical	Radio + MB	Mandibular R vs. superficial cervical R	Retropharyngeal R	HN0
JRT, SF, 8.9 years, 12.2 kg	Mammary Adenocarcinoma	52	6.5	CI	R thoracic mammary	Radio + MB	Inguinal L	Inguinal L	Negative
Boxer, IM, 7 years, 36 kg	MCT, Kiupel low grade	67	6.2	CI	R ventral cervical	Radio + MB	Superficial cervical R	Superficial cervical R	HN0
Mixed breed, SF, 13 years, 28.8 kg	MCT, Kiupel low grade	60	3	Marginal	L thigh	Radio+ MB + ICG	Inguinal L	Popliteal L	HN2
Mixed breed, SF, 16 years, 19.4 kg	MCT, Kiupel low grade	60	4	Marginal	R thoracic ventral	Radio+ MB + ICG	Axillary R	Axillary R and Accessory axillar R	HN0
French Bulldog, SF, 9 years, 10.2 kg	Subcutaneous MCT	35	NA	CI	R sternal	ICG	Axillary R	Axillar R and L	HN2
JRT, NM, 9 years, 12 kg	Subcutaneous MCT	90	4	Marginal	R sternal	ICG	Axillary R	Axillary R and Accessory axillary R	HN1
Deutscher Pinscher, NM, 7 years, 16.1 kg	MCT, Kiupel low grade	21	1	CI	L inguinal	ICG	Inguinal L	Inguinal R	HN2
Maltese, NM, 8 years, 4.6 kg	Subcutaneous MCT	38	6	CI	L thigh	ICG	Inguinal L	Inguinal L	HN2
Pug, SF, 5 years, 10.4 kg	MCT, Kiupel low grade	52	6	CI	L ventral abdomen	ICG	Inguinal L vs. R	Inguinal L and axillary	HN3
Mixed breed, SF, 8 years, 24.6 kg	MCT, Kiupel low grade	38	4	CI	L axillary	ICG	Axillary L*	Axillary L and Superficial cervical L	HN2
Mixed breed, NM, 10 years, 30.3 kg	Subcutaneous MCT	42	5	CI	Scrotum	ICG	Inguinal L vs. R	Inguinal L and R	HN2
Australian Shepherd, IM, 8 years, 24.6 kg	MCT, Kiupel low grade	100	5	Marginal	L scrotum	ICG	Inguinal L	Inguinal L	HN0
JRT, NM, 8 years, 10 kg	MCT, Kiupel low grade	42	1.5	CI	L inguinal fold	ICG	Inguinal L	Inguinal L	HN0
Chihuahua, SF, 9 years, 2.4 kg	Subcutaneous MCT	42	2	Marginal	Perineal L	ICG	Inguinal L	Inguinal R	HN0

Note: SLN: sentinel lymph node; SLNB: sentinel lymph node biopsy; JRT: Jack Russell Terrier; SF: spayed female; IF: intact female; NM: neutered male; IM: intact male; MCT: mast cell tumor; CI: curative intent; L: left; R: right; Radio: radiopharmaceutical; MB: Methylene blue; ICG: indocyanine green; * enlarged RLN; HN0: non metastatic lymph node; HN1: pre-metastatic lymph node; HN2 early metastatic lymph node; HN3: overt metastatic lymph node (based on Weishaar et al., 2014 [24]).

## Data Availability

The data that support the findings of this study are available from the corresponding author upon reasonable request.
